# An inflammatory memory and angiogenic self-assembling nanofiber hydrogel scaffold seeded with *Akkermansia muciniphila* to accelerate the healing of diabetic ischemic ulcers†

**DOI:** 10.1039/c8ra01662c

**Published:** 2018-05-14

**Authors:** Panke Cheng, Liyang Yao, Xiaolong Chen, Xingxing Su, Xuejiao Su, Qiang Huang, Chunli Hou

**Affiliations:** Department of Anatomy, Third Military Medical University Gao Tan Yan Street, Shaping Ba District Chongqing 400038 China houchunliwf@163.com; Department of Orthopaedics, Traditional Chinese Medicine Hospital Shaping Ba District Chongqing 400030 China

## Abstract

Refractory ulcers are a major challenge in the treatment of a diabetic foot, because of the immunodeficient, ischemic and high-glucose microenvironment. Inflammatory memory peptides, which were extracted from the immune mediator absent in melanoma 2 (AIM2), could effectively improve the immunodeficient microenvironment and special angiogenic peptides could effectively promote angiogenesis. Moreover, the gut flora *Akkermansia muciniphila* (*A. muciniphila*) participates in diabetic metabolism and could decrease high-glucose levels. In this research, a polypeptide skeleton (PPS) was synthesized based on 3,4-dihydroxyphenylalanine (DOPA) and peptides, forming the hydrophilic and hydrophobic parts. Inflammatory memory peptides and angiogenic peptides were synthesized and conjugated with the PPS, which then formed an anisotropic hydrogel through the self-assembling of β-sheet peptides based on hydrophobicity and DOPA oxidation. *A. muciniphila* was seeded into the hydrogel and transported into diabetic ischemic ulcers through subcutaneous injection, and the healing of diabetic ischemic ulcers was promoted. The inflammatory memory peptides were released based on the *A. muciniphila* enzyme response, and they firstly improved the immunity of the local surroundings. Then, the angiogenic peptides were also released through irradiation and they promoted angiogenesis. Additionally, the transported *A. muciniphila* could decrease the local glucose levels and spontaneously regress once the diabetic ischemic ulcers had healed. *A. muciniphila* combined with a functional polypeptide hydrogel may be a novel strategy for diabetic ischemic ulcer treatment.

## Abstract 2

In this research, a polypeptide skeleton was synthesized based on 3,4-dihydroxyphenylalanine (DOPA) and conjugated with inflammatory and angiogenesis peptides, combined with *A. Muciniphila* as a novel strategy for diabetic ischemic ulcer treatment.

## Graphical abstract

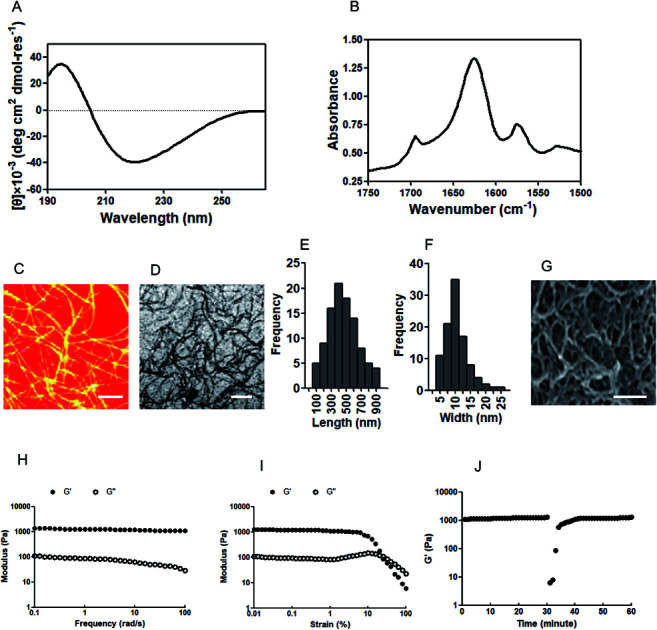

## Introduction

Refractory ulcers are often accompanied by peripheral vascular lesions, which eventually cause ischemia in a diabetic foot and the deterioration of the diabetic ischemic ulcers.^1^ Because of their poor local microenvironment with immunodeficiency, ischemia and high-glucose levels, diabetic ischemic ulcers have been hard to cure.^2^ According to pathological changes, trying to enhance the microenvironment by immune improvement, angiogenesis and decreasing glucose is the best solution for the healing of diabetic ischemic ulcers.

Previous reports have demonstrated that absent in melanoma 2 (AIM2) is a pyrin-HIN protein which binds to cytosolic double-stranded DNA and then activates the inflammasome, and the inflammasome receptor of AIM2 can effectively induce protective immune responses.^3,4^ This is called an inflammatory memory molecule. Although both immune improvement and angiogenesis are important for the healing of diabetic ischemic ulcers, immune improvement is a prerequisite; angiogenesis can maximize healing depending on the immune improvement.^5,6^

*Akkermansia muciniphila* (*A. muciniphila*) is a gut microbiota which could decrease glucose levels, trigger the immune response and inhibit cancer progress.^7^ Although *A. muciniphila* are beneficial for health, if *A. muciniphila* grows in the tissue or cytoplasm, the tissue or cell will suffer from catastrophic damage.^8^ Regarding the rule of combating poison with poison, whether damaged tissue such as diabetic ischemic ulcers can be protected by *A. muciniphila* is unknown but interesting.

*A. muciniphila* growth needs a suitable scaffold, and previous reports have demonstrated that hydrogel is a perfect growth matrix with injectable, biocompatible and biodegradable characteristics.^9^ In addition, peptide hydrogel is used more and more widely, because it can be assembled easily under mild controlled conditions, and due to its advantages in cell or drug encapsulation and delivery. Moreover, peptide hydrogel may consist of functional peptide sequences, such as inflammatory memory and angiogenic peptides, and the bio-function of peptide hydrogel is then further promoted.^10,11^ Although functional peptide sequences can be effectively conjugated with the peptide hydrogel, the functional peptide sequence effects just depend on the free peptide sequences. Moreover, different functional peptides need to be released at different times and so the controlled release method is necessary for functional peptide release.

In this study, inflammatory memory peptides were extracted from the *Aim2* protein, hydrophilic and hydrophobic peptides were synthesized according to the solid-phase peptide synthesis methodology, and the inflammatory memory peptides and angiogenic peptides were conjugated with the hydrophilic and hydrophobic peptides based on the enzymatic decomposition linker and photodegradation linker respectively, and then forming the form anisotropic hydrogel through the self-assembling of β-sheet peptides according to their hydrophilic or hydrophobic characteristics and DOPA (3,4-dihydroxyphenylalanine) oxidation. The gut microbiota *A. muciniphila* was seeded into the hydrogel, and injected into the diabetic ischemic ulcer tissues, the inflammatory memory peptides were released through the function of *A. muciniphila* enzymes, and the angiogenic peptides were released using irradiation. In the diabetic ischemic ulcer region, the protective immune response was enhanced by the inflammatory memory peptides, the blood supply was promoted by the angiogenic peptides, and the local glucose level was reduced by the function of *A. muciniphila*. Based on the complex functions of the hydrogel, the healing of diabetic ischemic ulcers was effectively accelerated.

## Results and discussion

### Synthesis of the modified PPS

**Fig. 1:**
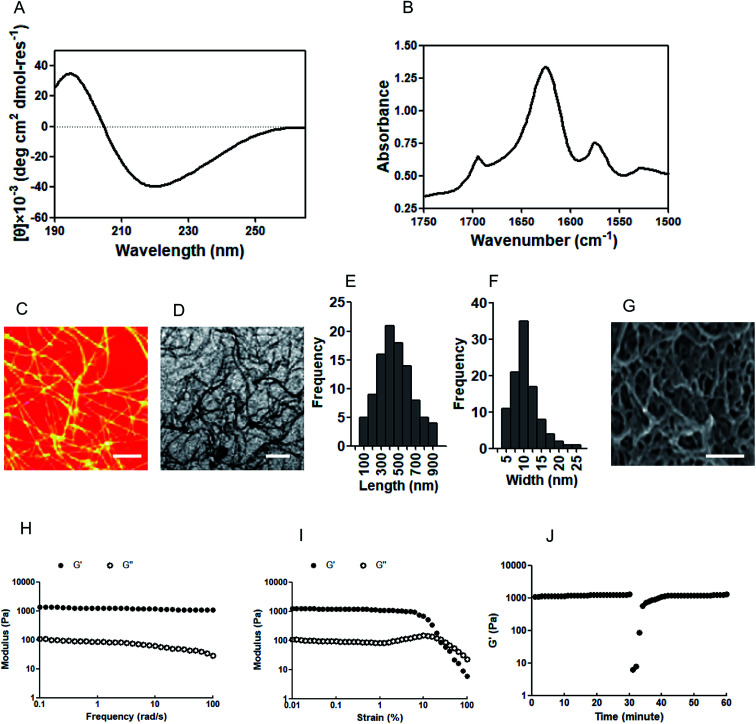
Physicochemical characteristics of the hydrogel. (A) A CD spectrum of hydrogel showing a minimum between 210 nm and 230 nm, suggesting a β-sheet formation. (B) FTIR spectrum of the amide I region showing the peaks of an antiparallel β-sheet at 1630 cm^−1^ and 1695 cm^−1^. (C) AFM images of peptide self-assembled peptide nanofibers with scale bars for 100 nm. (D) TEM images of peptide self-assembled peptide nanofibers with scale bars for 50 nm. The statistical results of (E) the lengths and (F) the widths of self-assembled peptide nanofibers. (G) SEM images of the hydrogel with scale bars for 500 nm. The *G*′ and *G*′′ with shear thinning detection of the hydrogel through oscillatory shear rheology assays at (H) strain rates and (I) frequency oscillation. (J) The recovery detection of hydrogel at a high shear rate.

**Fig. 2:**
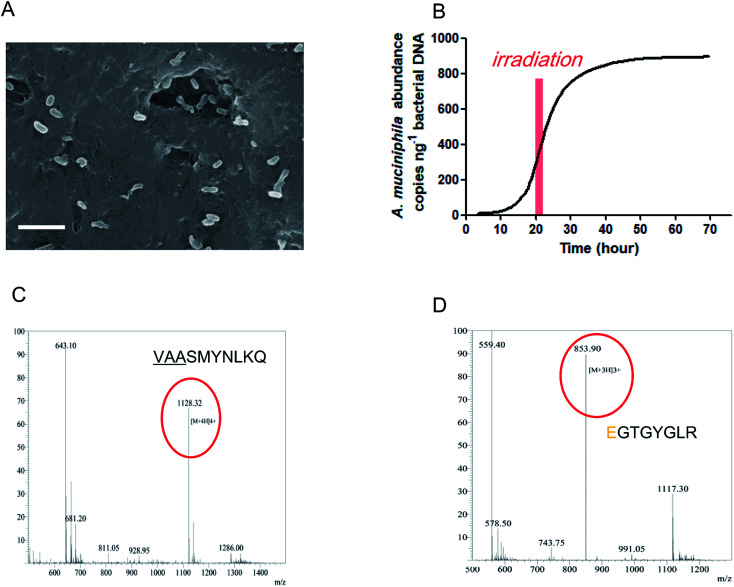
Biological characteristics of the AMIH. (A) A SEM image of the AMIH with scale bars for 10 μm. (B) The concentration of *A. muciniphila* in the AMIH at different time points using qPCR analysis, with irradiation lasting 2 hours. The mass spectral assays of AMIH products for (C) inflammatory memory functional peptide detection after *A. muciniphila* seeding for 3 days and (D) angiogenic peptide detection after *A. muciniphila* seeding for 5 days and undergoing irradiation.

**Fig. 3:**
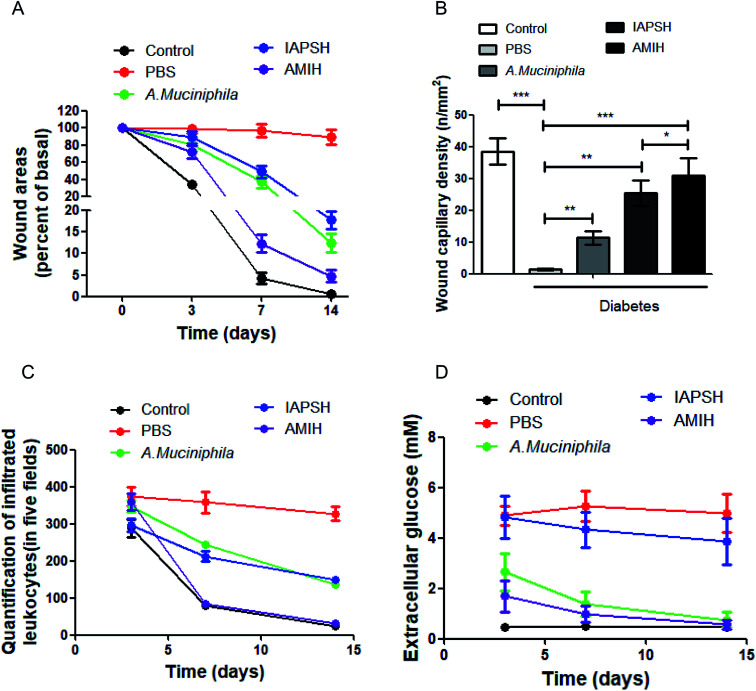
The effect of the AMIH on the healing of diabetic ischemic ulcers. The statistical results of (A) wound area, (B) wound capillary density, (C) leukocyte infiltration and (D) local extracellular glucose levels. *P < 0.05; **P < 0.01; ***P < 0.005.

In order to obtain its self-assembling characteristic, the polypeptide skeleton (PPS) was designed as “EXGTGXGSVXGSVXGSVXGTGXE” (the Xs represent DOPA), including the hydrophilic and hydrophobic residues (Fig. 1, ESI†). PPS was synthesized through a solid phase peptide synthesis method and the results of high performance liquid chromatography (HPLC) (Fig. 2, ESI†) and the mass spectra (Fig. 3, ESI†) indicated that PPS was successfully synthesized. Moreover, PPS was modified by amine and azide in different residues (Fig. 4, ESI†).

### Synthesis of inflammatory memory and angiogenic peptides

Previous reports have demonstrated that *A. muciniphila* can produce more than 60 enzymes for mucin degradation, such as glucosidase, sulphate esterase and sialidase, and the special peptide sequence “VAA” is the recognized and hydrolytic site of *A. muciniphila* enzymes.^12,13^ When the body is suffering damage, the protective immune response is quickly activated by inflammatory memory related molecules and triggers the immune cascade process.^14^ In the inflammatory memory response, AIM2, which is an activator of inflammasomes, can activate the protective immune response. The functional domain of AIM2 is a series of polypeptide sequences and the core sequence is “SMYNLKQ”.^15^ By combining *A. muciniphila* enzyme response peptides and inflammatory memory peptides, we designed and synthesized the inflammatory memory functional peptide “TVAASMYNLKQ”, where the sequence of VAA is the *A. muciniphila* enzyme response site. The results of HPLC (Fig. 5, ESI†) and the mass spectra (Fig. 6, ESI†) suggested that the inflammatory memory functional peptide was successfully synthesized.

### Synthesis of angiogenic peptides

The compound 2-amino-5-(4-(carboxymethoxy)-7-nitroindolin-1-yl)-5-oxopentanoic acid (ACNOA) can be decomposed into two separate molecules, by irradiation, including glutamic acid (E). ACNOA was synthesized through a special chemical synthesis process (Fig. 7, ESI†). The ^1^H NMR spectra (Fig. 8, ESI†), ^13^C NMR spectra (Fig. 9, ESI†) and mass spectra (Fig. 10, ESI†) confirmed that ACNOA was successfully synthesized. Moreover, we have performed HPLC to purify ACNOA (Fig. 11, ESI†). A previous report has demonstrated that a special peptide sequence, “EGTGYGLR”, could promote angiogenesis under physiological and pathological conditions.^16^ In order to synthesize an angiogenic polypeptide with irradiation decomposition, the special sequence “BLOGTGYGLR” (“B” represents l-propargylglycine and “O” represents ACNOA) was designed and synthesized. The results of the mass spectra (Fig. 12, ESI†) and HPLC (Fig. 13, ESI†) indicated that the angiogenic peptide “BLOGTGYGLR” was successfully synthesized.

### Inflammatory memory and angiogenic peptides conjugated with a PPS to form IAPS

In the treatment of diabetic ischemic ulcers, immune mediation is vital for local microenvironment improvement, following angiogenesis and cell growth, so the non-invasive and controlled release of therapeutic factors is useful for wound healing.^17,18^ There are studies which have demonstrated that enzyme or irradiation decomposition was effectively a non-invasive method for special molecule degradation, especially for peptide separation.^19,20^ We conjugated inflammatory memory peptides and angiogenic peptides with a PPS at different sites (IAPS). The inflammatory memory peptide “VAASMYNLKQ” could be released by IAPS by the *A. muciniphila* enzyme response, and the angiogenic peptide “EGTGYGLR” could be released by IAPS by irradiation (Fig. 14, ESI†). Moreover, the results of the mass spectra (Fig. 15, ESI†) and HPLC (Fig. 16, ESI†) indicated the successful formation of IAPS.

### The formation of IAPSH

**Scheme 1:**
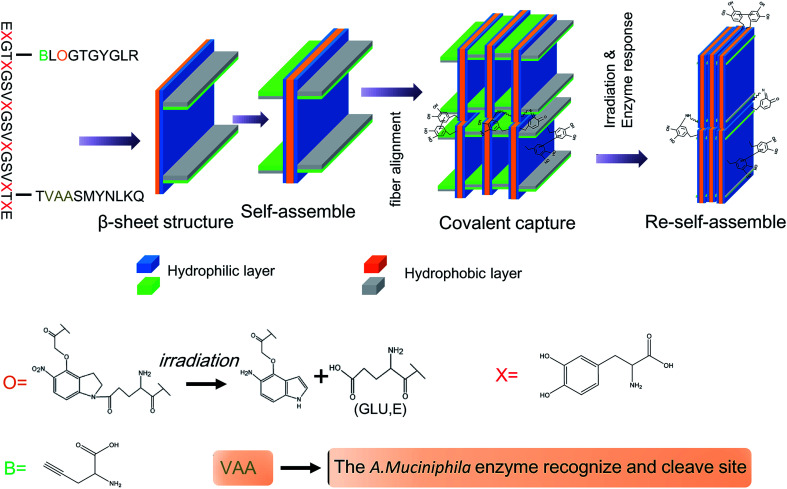
Schematic of IAPSH formation and response.

The amino acids of the IAPS have a high propensity to form a β-sheet structure, and the β-sheets can then self-assemble into bilayers of antiparallel β-sheets due to the hydrophilic and hydrophobic residues. Furthermore, bilayers of antiparallel β-sheets could further self-assemble into nanofibers to form an anisotropic hydrogel (IAPSH) based on the oxidative cross-linking of DOPA and hydrophilic and hydrophobic residues. Although the functional peptide sequences of IAPSH were released according to the effects of *A. muciniphila* enzymes and irradiation, residual sequences could self-assemble into new nanofibers and form a new anisotropic hydrogel based on the oxidative cross-linking of DOPA (PSH) (Scheme 1). A previous report has demonstrated that DOPA modified amino acids display a noninduced transition from spherical assemblies into nanofibrils, followed by sol–gel transition, nanotube formation *via* intermediate assembly, and crystallization within the gel, providing a spontaneous coordinated structural transition model.^21^ Functional peptides were conjugated with DOPA modified amino acids in our research, and these self-assembled into nanofibers based on hydrophobic associations and the oxidative cross-linking of DOPA, and further formed the hydrogel.

### The characteristics of IAPS and IAPSH

A circular dichroism (CD) assay was performed to clarify the secondary structure of the IAPS. Previous reports have demonstrated that CD spectrum results with a minimum around 218 nm indicate a β-sheet conformation.^22^ In our research, the CD spectrum results suggest that the IAPS has a β-sheet secondary structure with a maximum around 195 nm and minimum around 218 nm (Fig. 1A). Fourier transform infrared spectroscopy (FTIR) was performed to further confirm the secondary structure of the IAPS, and the results indicate that the amide I band (the 1625 cm^−1^ peak) is consistent with a β-sheet structure and moreover, the peak at 1695 cm^−1^ further indicates the formation of an antiparallel β-sheet (Fig. 1B). In order to detect the nanostructure of the IAPSH, the materials were dehydrated *via* critical point drying, and then an atomic force microscope (AFM) assay and a transmission electron microscopy (TEM) assay were performed to show the morphology of the IAPSH. The results of both the AFM assay (Fig. 1C) and TEM assay (Fig. 1D) indicated that the IAPS self-assembled into fibrils, with the lengths distributed from 100 nm to 900 nm (Fig. 1E) and the widths ranging from 5 nm to 25 nm (Fig. 1F). Moreover, the IAPSH was dehydrated *via* critical point drying and observed using scanning electron microscopy (SEM) and the results suggested that the IAPSH had a highly porous structure based on the connected network of IAPS fibrils (Fig. 1G). Moreover, rheological characterization was performed to detect the mechanical properties of the IAPSH and the results suggested that the IAPS could form the IAPSH at 20 mM in water and have storage moduli with approximately 1200 Pa, and the mechanical properties of the IAPSH were almost unaffected by the frequency of the oscillatory strain in a frequency sweep (Fig. 1H). Furthermore, the IAPSH results suggested plastic strain shearing at about 20% strain (Fig. 1I). In order to confirm the suitability for delivery and syringe aspiration, the IAPSH was sheared at 100% strain for 1 min and then returned to 1% strain. Compared with its original mechanical rigidity, the IAPSH could recover more than 95% within seconds (Fig. 1J). These results indicated that the IAPSH could be aspirated and dispensed through small needles, and form a hydrogel immediately. In addition, once the *A. muciniphila* enzyme response was triggered, inflammatory memory functional peptides were released from the IAPS (APS) and the residual peptides could form the new hydrogel (APSH). After the *A. muciniphila* enzyme response and once the irradiation reaction was performed, the residual angiogenic peptides were also released from the APS (PS) and interestingly, the residual peptides could also form the new hydrogel (PSH). The physical characterization of the APSH (Fig. 17, ESI†) and PSH (Fig. 18, ESI†) was also performed. Although the APS also had a β-sheet secondary structure (Fig. 17A, ESI†), the antiparallel β-sheets disappeared when the inflammatory memory functional peptides were released (Fig. 17B, ESI†); the APS could also self-assemble into fibrils (Fig. 17C and D, ESI†) with lengths of 50 nm to 400 nm (Fig. 17E, ESI†) and widths of 2 nm to 16 nm (Fig. 17F, ESI†). The density of the porous structure of the APSH was lower than the IAPSH (Fig. 17G, ESI†). The results of the rheological characterization indicated that the APSH had gel liquefaction at about 30 rad s^−1^ (Fig. 17H, ESI†), and plastic strain shearing at about 20% strain (Fig. 17I, ESI†), moreover, when the APSH was sheared at 100% strain for 1 min and then returned to 1% strain, the APSH could recover greater than 95% of its original mechanical rigidity within minutes, but the recovery time was longer than that for the IAPSH (Fig. 17J, ESI†). When the APSH received irradiation, angiogenic peptides were released from the APS (PS) and like the APS, the PS also had a β-sheet secondary structure (Fig. 18A, ESI†) and antiparallel β-sheets (Fig. 18B, ESI†). Moreover, the PS could re-self-assemble into fibrils (Fig. 18C and D, ESI†) with lengths of 200 nm to 1800 nm (Fig. 18E, ESI†) and widths of 5 nm to 45 nm (Fig. 18F, ESI†). The hydrogel structure of the PSH was dense with not much porous structure (Fig. 18G, ESI†). Although the mechanical properties of the PSH were almost unaffected by the frequency of the oscillatory strain in a frequency sweep (Fig. 18H, ESI†) and plastic strain shearing at about 20% strain (Fig. 18I, ESI†), the PSH could not recover greater than 95% of its original mechanical rigidity when shearing the hydrogel (Fig. 18J, ESI†).

### Characteristics of the IAPSH with *A. muciniphila* growth

According to the above results, the IAPSH was a perfect scaffold for *A. muciniphila* growth. In this research, we seeded the *A. muciniphila* into the IAPSH (AMIH), and the SEM results indicate that *A. muciniphila* grew well in the IAPSH (Fig. 2A). In order to accurately detect the activity of *A. muciniphila*, a Q-PCR assay was performed to detect the proliferation of *A. muciniphila*. The results suggested that *A. muciniphila* was growing exponentially after *A. muciniphila* seeding for 15 h, moreover, although irradiation promoted the release of angiogenic peptides, the growth of *A. muciniphila* was not affected during the irradiation period (Fig. 2B). Besides, in order to confirm the effect of *A. muciniphila* enzymes on the release of inflammatory memory functional peptides, the products of the AMIH were collected after *A. muciniphila* seeding for 3 days, and when a mass spectral assay was performed, the results indicated that the inflammatory memory functional peptide “VAASMYNLKQ” was released from the IAPSH (Fig. 2C). Furthermore, the residual AMIH was irradiated after *A. muciniphila* seeding for 5 days and the products were collected and a mass spectral assay was performed. The results suggested that the angiogenic peptide “EGTGYGLR” was released (Fig. 2D). The inflammatory memory functional peptides and angiogenic peptides were successfully released from the AMIH, becoming free peptides which could further play roles in immune mediation and angiogenic promotion.

### Treatment of the AMIH on diabetic ischemic ulcers

In order to clarify the effect of the AMIH on diabetic ischemic ulcers, C57 mice were used to establish a diabetic ischemic ulcer model. All animal procedures were performed in accordance with the Guidelines for Care and Use of Laboratory Animals of Third Military Medical University and the experiments were approved by the Animal Ethics Committee of Third Military Medical University. The mice were treated with different subcutaneous injection strategies, including the injection of PBS, *A. muciniphila*, IAPSH and AMIH. Moreover, normal C57 mice went through the same operation procedure as the control group. In the IAPSH and AMIH treated groups, the mice were given the irradiation on the 5^th^ day. At 0, 3, 7 and 14 days, the healing of the wounds was photographed and recorded. The results suggested that both the *A. muciniphila* and IAPSH treatments could promote the healing of diabetic ischemic ulcers, and their effects were almost the same; interestingly, when the mice received the AMIH treatment, the effect on the healing of the wound was the best, which was almost the same as the recovery of the normal mice (Fig. 3A and Fig. 19, ESI†). The results indicated that the AMIH could effectively promote the healing of diabetic ischemic ulcers. Moreover, for further evaluation of the effect of the AMIH on the recovery of diabetic ischemic ulcers, we collected the ulcer tissues and performed an immunofluorescence assay for the CD31 detection marker, and counted the number of capillaries. Due to the improvement of the immune environment and the effect of the released angiogenic peptides, the density of capillaries was highest in the AMIH-treated group; as the angiogenic peptides were released after irradiation in the IAPSH-treated group, the density of capillaries also increased significantly (Fig. 3B and Fig. 20, ESI†). The improvement of the immune environment is vital for the healing of wounds, and leukocyte infiltration can effectively reflect the immune response. Once the immune response is triggered, leukocytes accumulate and enhance the immunological cascade, then promote the healing of the wound. After inflammation has subsided the number of leukocytes decreases quickly; however, the continuous deterioration of the immune environment can increase the number of leukocytes, further deteriorate the healing of the wound and contribute to the refractory ulcer.^23,24^ According to the leukocyte infiltration detection in our research, the results indicate that the AMIH treatment could effectively mediate the immune environment based on the inflammatory memory peptides which were released by the AMIH depending on the *A. muciniphila* enzyme response. In the AMIH-treated group, the number of leukocytes was almost the same as the normal group; although the IAPSH contained the inflammatory memory peptides, *A. muciniphila* was not seeded in the IAPSH and no free inflammatory memory peptides were released, so the number of leukocytes in the IAPSH-treated group was more than in the AMIH-treated group (Fig. 3C and Fig. 21, ESI†). In diabetic ischemic ulcers, the local glucose level is high, which interferes with the recovery process of the ulcer and can lead to non-healing ulcers.^25^ Previous reports have demonstrated that *A. muciniphila* could effectively reduce blood glucose levels, but there has been no research on the effect of *A. muciniphila* on local extracellular glucose mediation.^26^ In this research, we detected local extracellular glucose levels in diabetic ischemic ulcers. The results indicated that treatment with *A. muciniphila* and AMIH could significantly decrease extracellular glucose levels, contributing to the improvement of ulcer local microenvironments, and then promoting the healing of diabetic ischemic ulcers (Fig. 3D). Although *A. muciniphila* could effectively mediate local extracellular glucose levels, *A. muciniphila* is bacteria and so only a limited quantity of *A. muciniphila* could fully play its biological function as too much *A. muciniphila* would be harmful for the body.^27^ In this study, we conducted *A. muciniphila* abundance detection, and the result indicated that the amount of *A. muciniphila* reached a peak on the 4^th^ day, lasted 6 days, then decreased quickly until zero (Fig. 22, ESI†). The above results indicated that the AMIH treatment could effectively promote the healing of diabetic ischemic ulcers. The reason for this is that the AMIH has multiple effects on the recovery of ulcers, including immune mediation which was based on the function of free inflammatory memory peptides, angiogenic promotion which was based on the effect of free angiogenic peptides, and glucose level meditation which depended on the function of *A. muciniphila*. Therefore, the AMIH has great potential for diabetic ischemic ulcer treatment in the future.

## Conclusion

As it is a difficult disease, a large number of people are still suffering from diabetic ischemic ulcers. However, there are no effective treatments for diabetic ischemic ulcers because of its complex pathological changes. In this research, we synthesized peptide hydrogel through complex functional peptide self-assembling based on hydrophilic and hydrophobic interactions as well DOPA oxidation. The gut flora *A. muciniphila* was seeded into the peptide hydrogel and formed the AMIH. In the animal experiments, the AMIH could significantly promote the healing of diabetic ischemic ulcers through angiogenesis promotion, immune mediation and local glucose level mediation. Moreover, the gut flora *A. muciniphila* in the AMIH could spontaneously regress after a period of AMIH injections in diabetic ischemic ulcers. Therefore, the reported treatment of diabetic ischemic ulcers in this research may be a novel and effective treatment in the future.

## Materials and methods

The complete detailed materials and methods are provided in the ESI.†

## Conflicts of interest

The authors confirm that there are no conflicts of interest.

## Abbreviations

AIM2: Absent in melanoma 2
*A. muciniphila*
: *Akkermansia muciniphila*
DOPA: 3,4-Dihydroxyphenylalanine
ACNOA: 2-Amino-5-(4-(carboxymethoxy)-7-nitroindolin-1-yl)-5-oxopentanoic acid
PPS: Polypeptide skeleton (PPS)
IAPS: Inflammatory memory peptide and angiogenic peptide conjugated with PPS
IAPSH: IAPS forming the hydrogel
APS: Inflammatory memory peptides released from IAPS
APSH: APS forming the hydrogel
PS: Angiogenic peptides released from APS
PSH: PS forming the hydrogel
AMIH: *A. muciniphila* seeded into the IAPSH

## Supplementary Material

RA-008-C8RA01662C-s001
