# Supplementary material for: An inflammatory memory and angiogenic self-assembling nanofiber hydrogel scaffold seeded with Akkermansia muciniphila to accelerate the healing of diabetic ischemic ulcers
Source: RSC Adv. 2018 May 14;8(31):17357–64. doi: 10.1039/c8ra01662c (PMC9080442; doi:10.1039/c8ra01662c)
Supplement: RA-008-C8RA01662C-s001 [file RA-008-C8RA01662C-s001.pdf]

Electronic Supplementary Material (ESI) for RSC Advances.
This journal is © The Royal Society of Chemistry 2018

Support information

Inflammatory memory and angiogenesis self-assembling nanofibers hydrogel scaffold seeded with *Akkermansia Muciniphila* accelerate the healing of diabetic ischemic ulcers

Panke Cheng[1], Liyang Yao[1], Xiaolong Chen[1], Xingxing Su[1], Xuejiao Su[1], Qiang Huang[2] and Chunli Hou[1 †]

1. Department of Anatomy, Third Military Medical University, Chongqing 400038, China

2. Department of Orthopaedics, Traditional Chinese Medicine Hospital, Shaping Ba District, Chongqing 400030, China

†Correspondence to: Chunli Hou, Department of Anatomy, Third Military Medical University, Gao Tan Yan Street, Shaping Ba District, Chongqing, 400038, China

E-mail: houchunliwf@163.com

Support figures

Peptide sequence: EXGTGXGSVXGSVXGSVXGTGXE

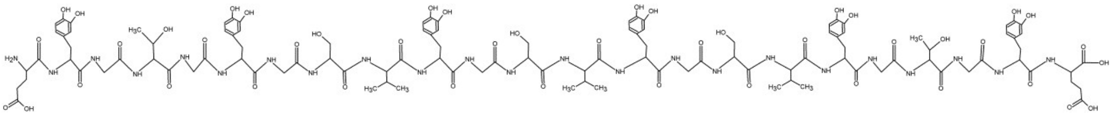

Fig 1. The chemical structural formula of polypeptide skeleton (PPS)

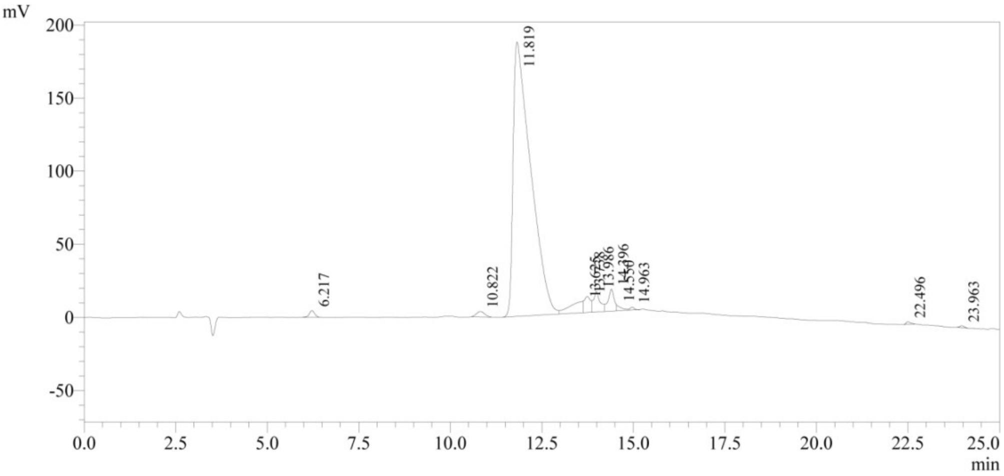

Fig2. The HPLC of PPS.

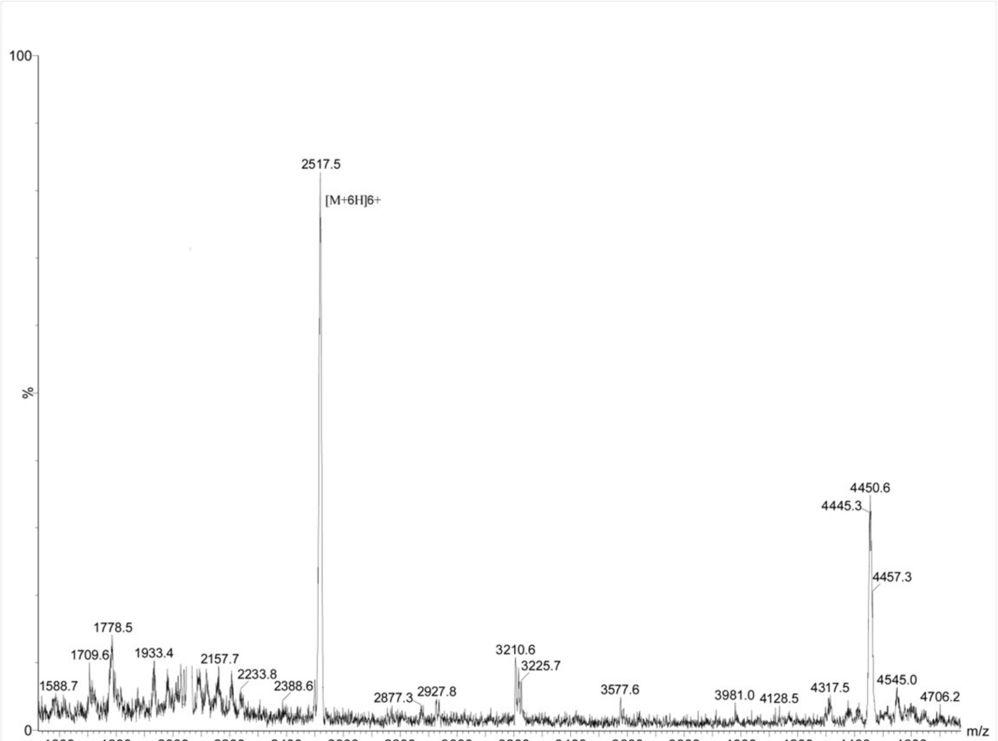

Fig3. The MALDI-TOF mass spectra of PPS.

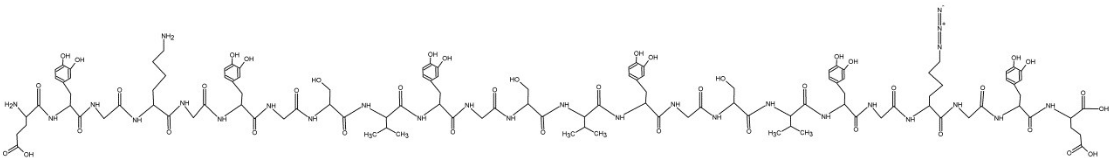

Fig4. The chemical structural formula of modified PPS

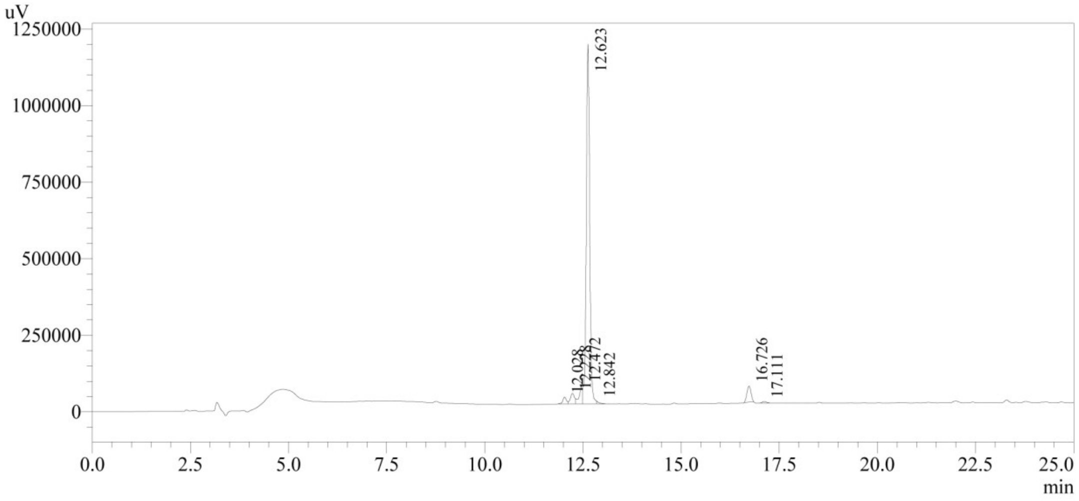

Fig5. The HPLC of angiogenesis functional peptide sequences.

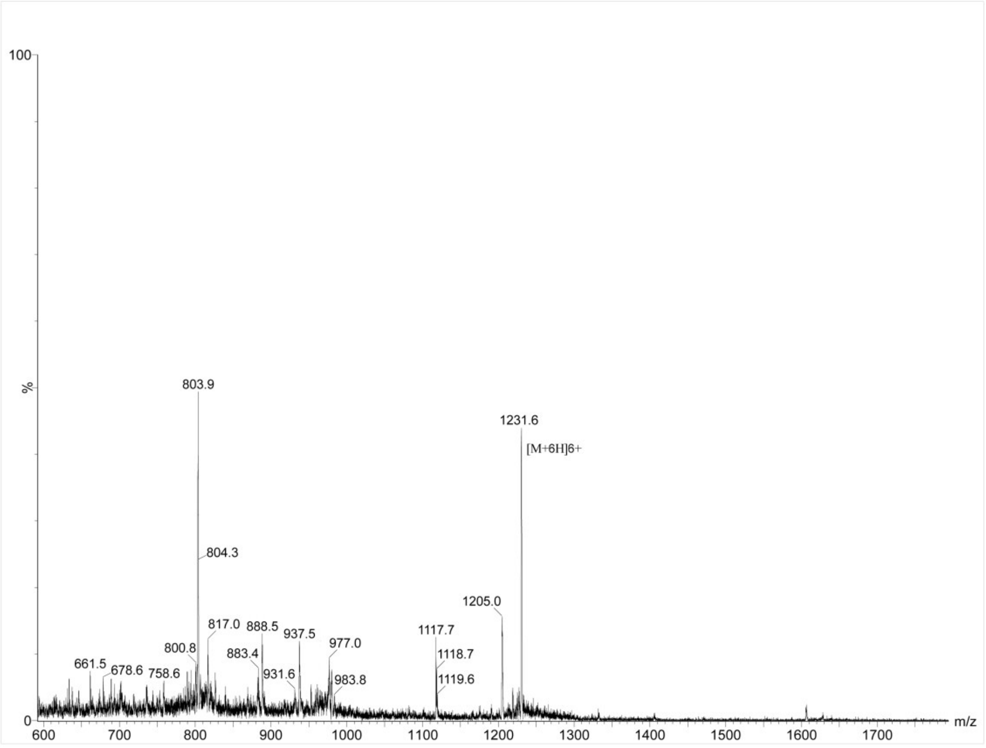

Fig6. The MALDI-TOF mass spectra of angiogenesis functional peptide sequences.

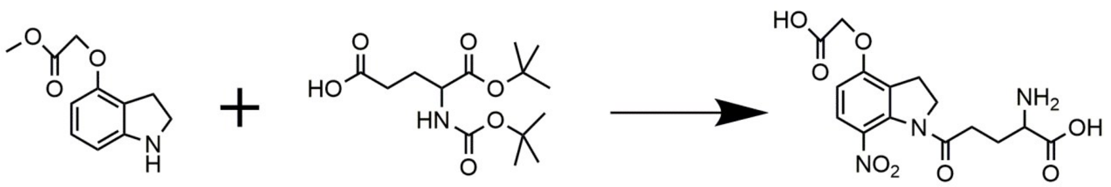

Fig7. The process of 2-amino-5-(4-(carboxymethoxy)-7-nitroindolin-1-yl)-5-oxopentanoic acid (ACNOA) synthesis.

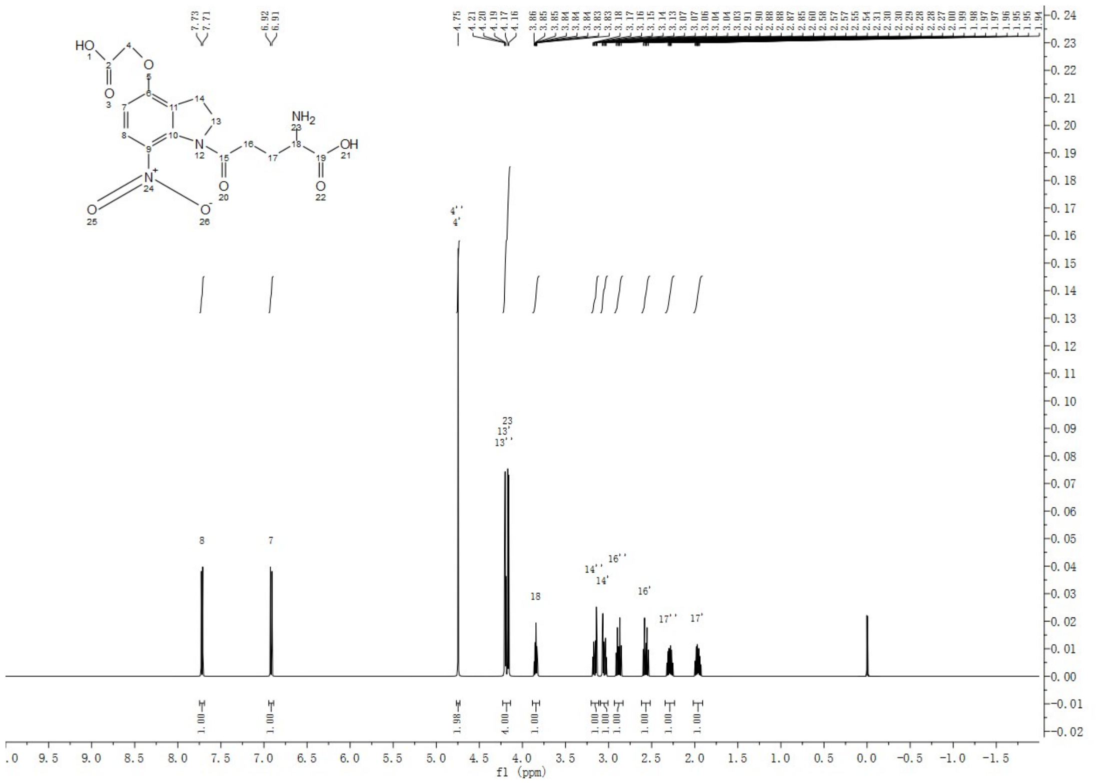

Fig8. The ^{1}H NMR spectra of ACNOA.

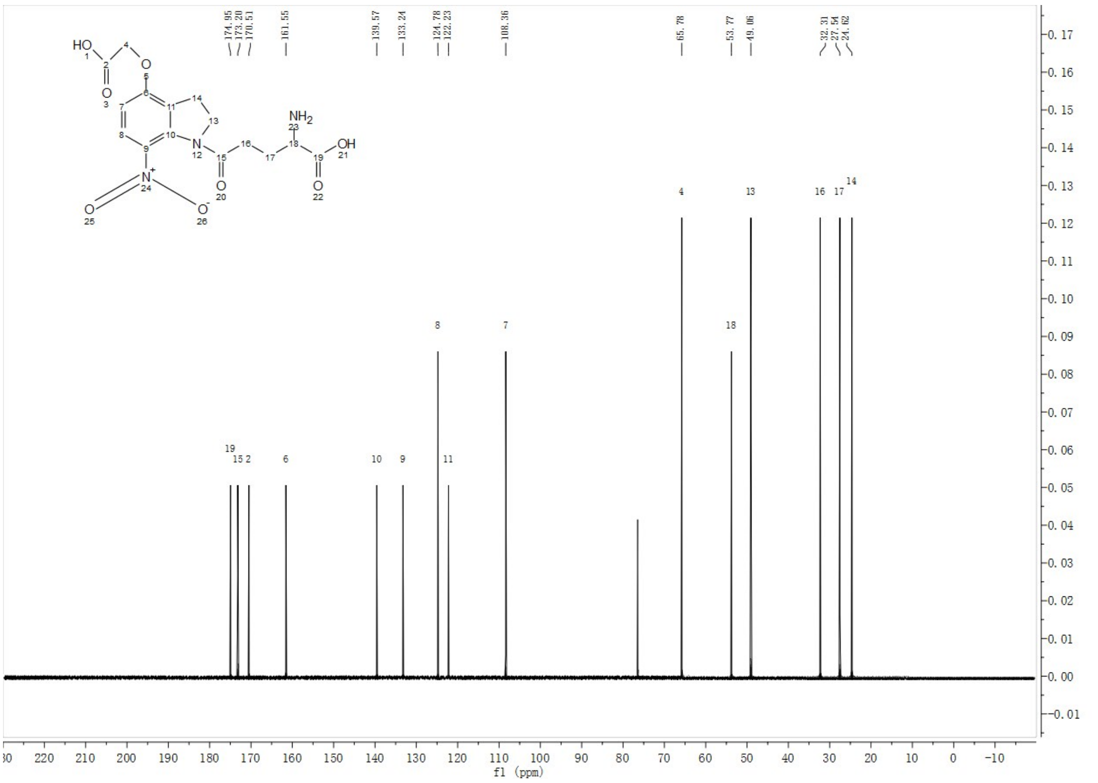

Fig9. The ^{13}C NMR spectra of ACNOA.

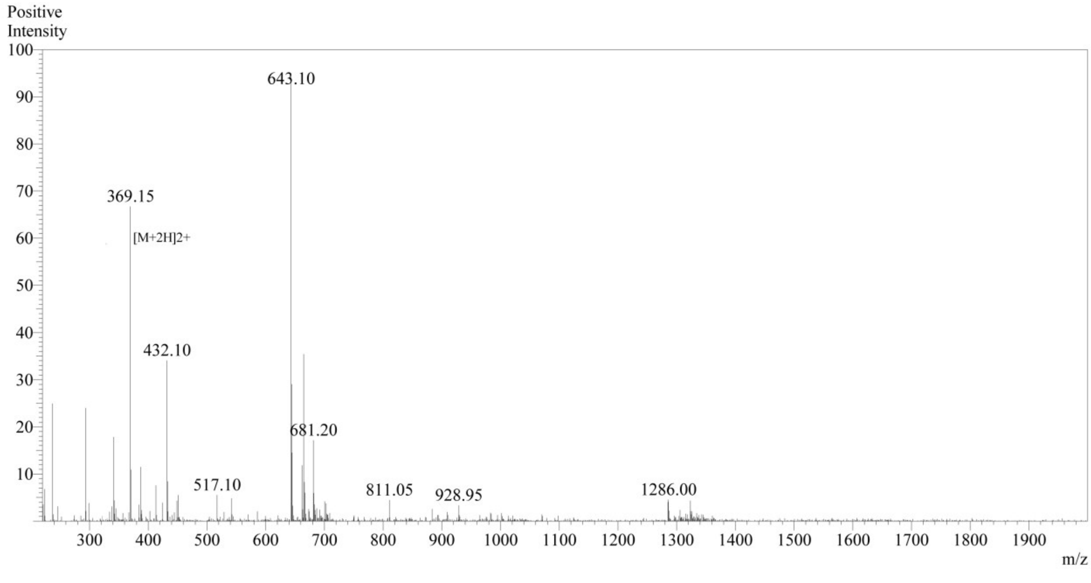

Fig10. The mass spectra of ACNOA.

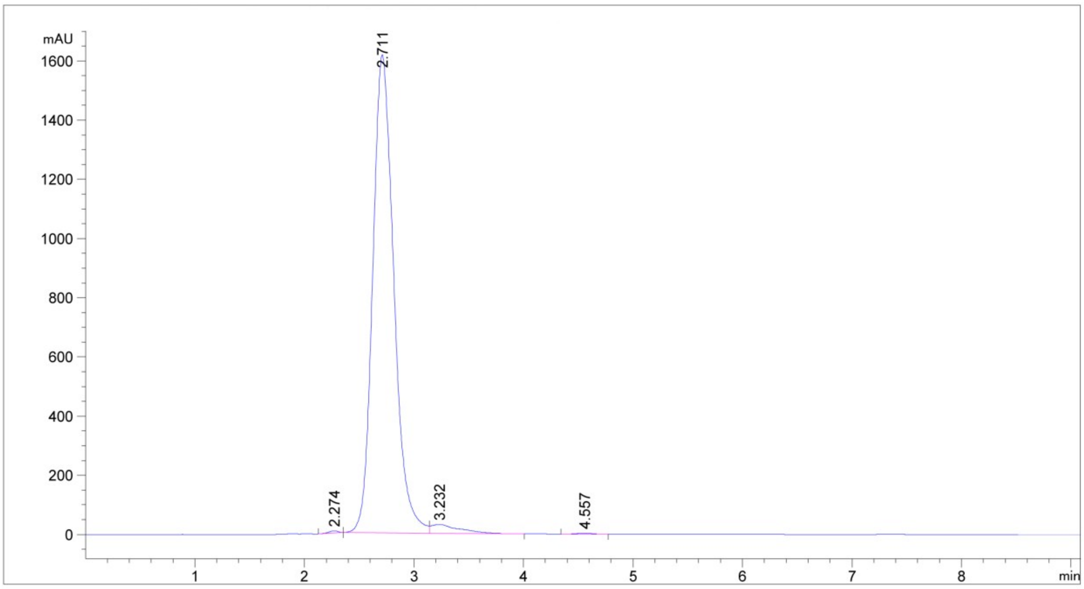

Fig11. The HPLC of ACNOA.

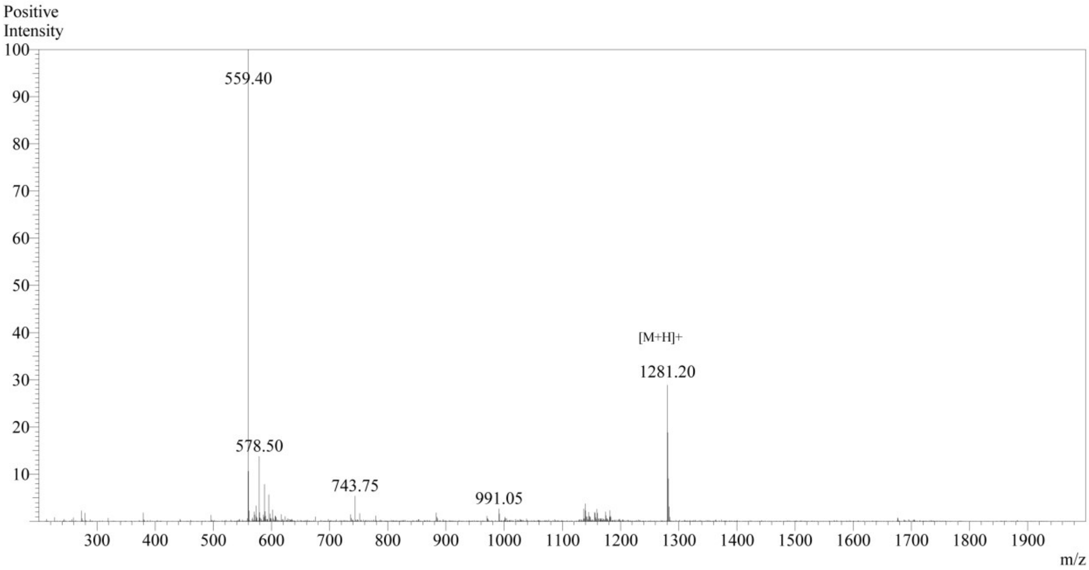

Fig12. The mass spectra of angiogenesis polypeptide.

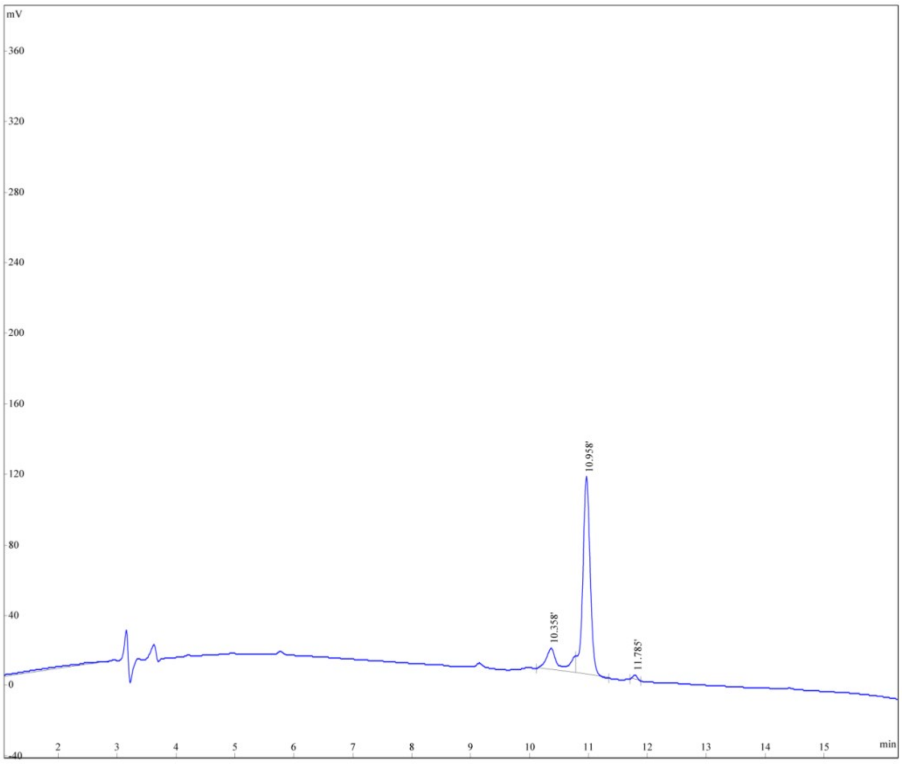

Fig13. The HPLC of angiogenesis polypeptide.

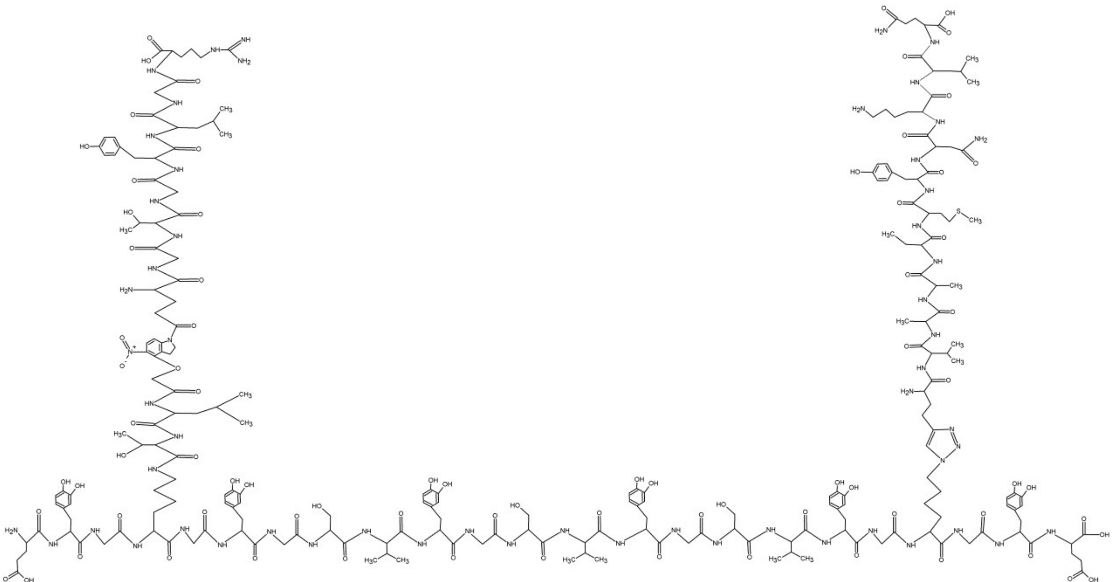

Fig14. The chemical structural formula of PPS conjugated with inflammatory memory functional peptide and angiogenesis polypeptide at different site (IAPS).

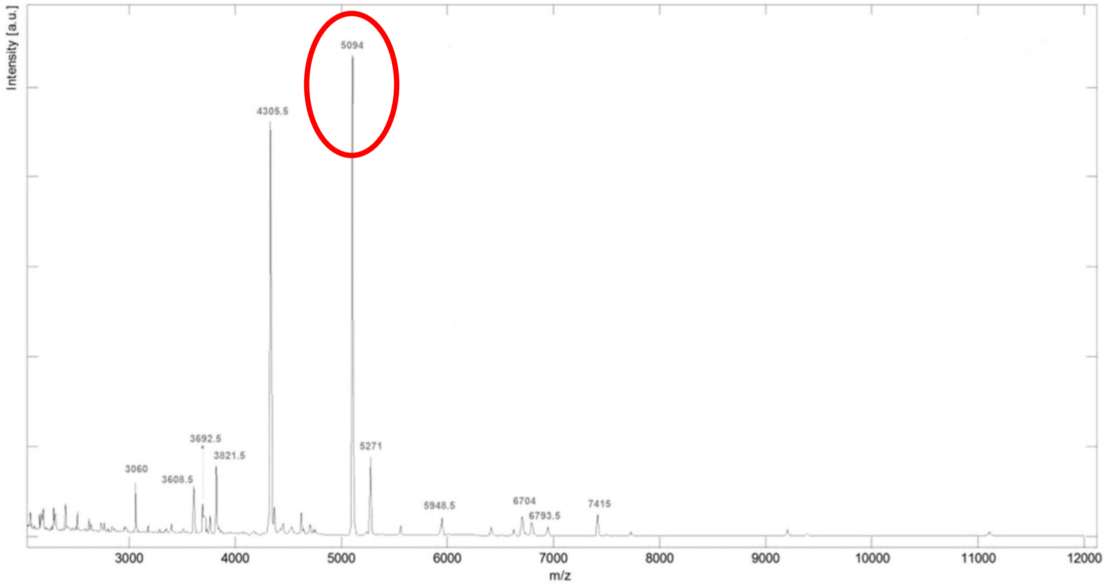

Fig15. The mass spectra of IAPS.

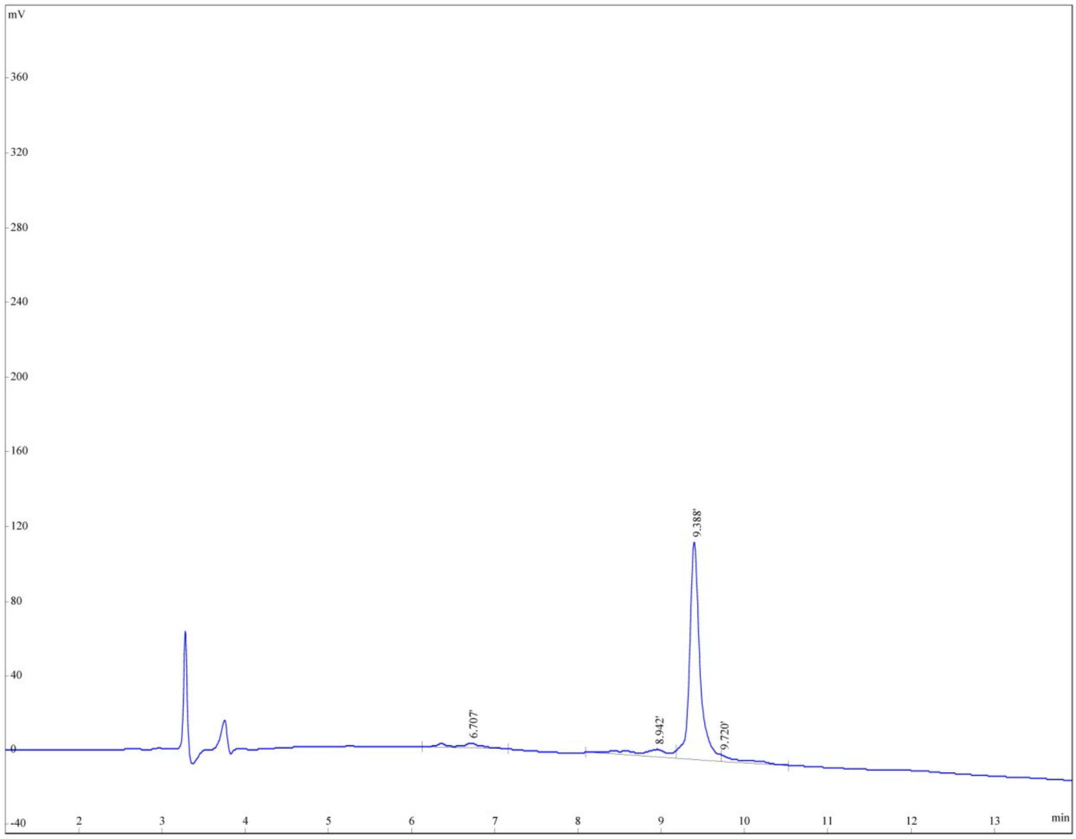

Fig16. The HPLC of IAPS.

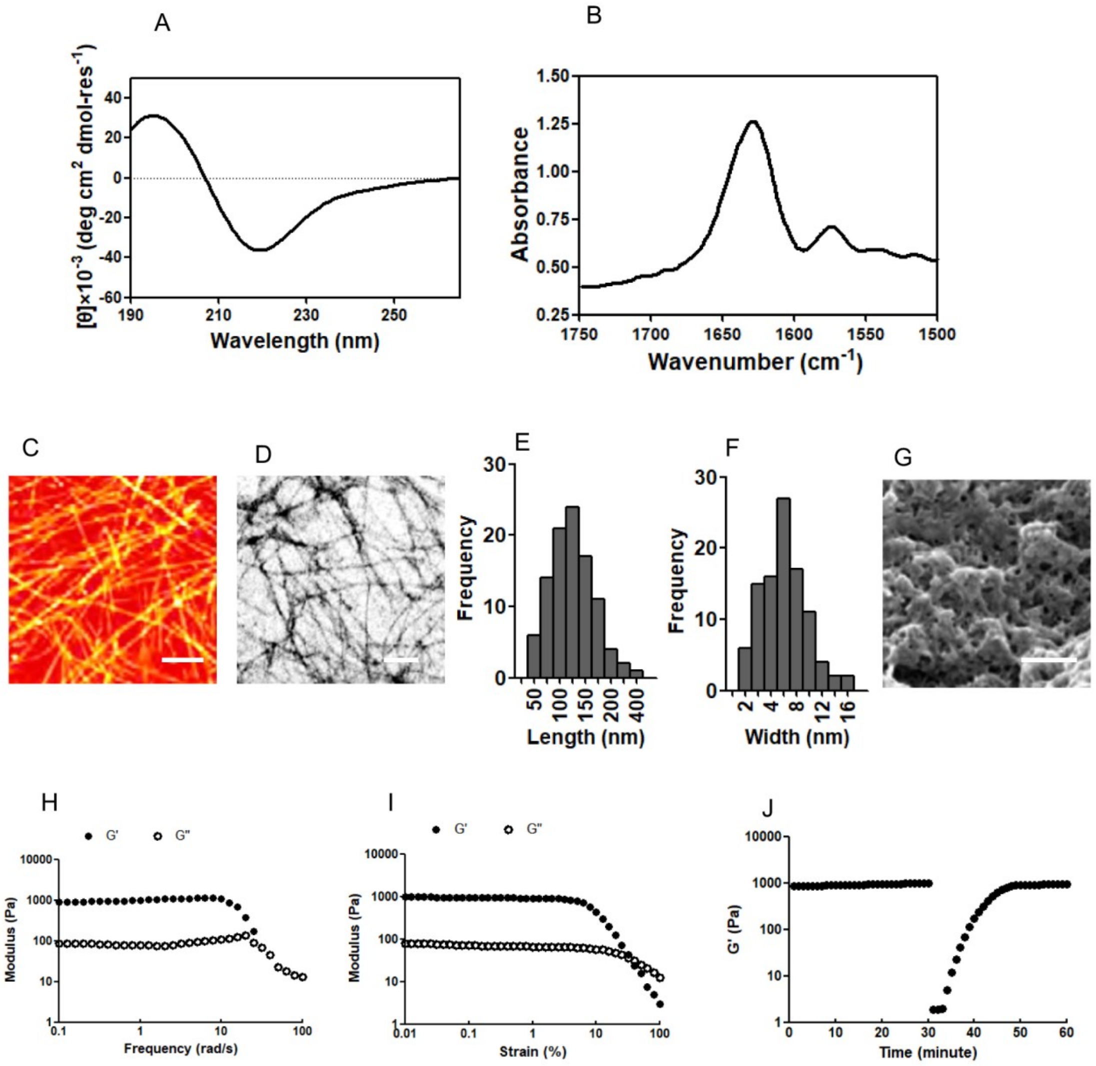

Fig17. Physicochemical characteristics of hydrogels after *A.Muciniphila* enzyme response. (A) CD spectrum of hydrogels show the minima between 210 nm and 230 nm, suggesting the β-sheet formation. (B) FTIR spectrum of the amide I region showed the peaks of β-sheet at 1630 cm^{-1}. (C) AFM images of peptide self-assembled peptide nanofibers, scale bars for 100 nm. (D) TEM images of peptide self-assembled peptide nanofibers, scale bars for 50 nm. The statistical results of (E) the lengths and the (F) width about self-assembled peptide nanofibers. (G) SEM images of hydrogels, scale bars for 500 nm. The G′ and G″with shear thinning detection of hydrogels through oscillatory shear rheology assays at (H) strain rates and (I) frequency oscillation. (J) The recovery detection of hydrogels from high shear rate.

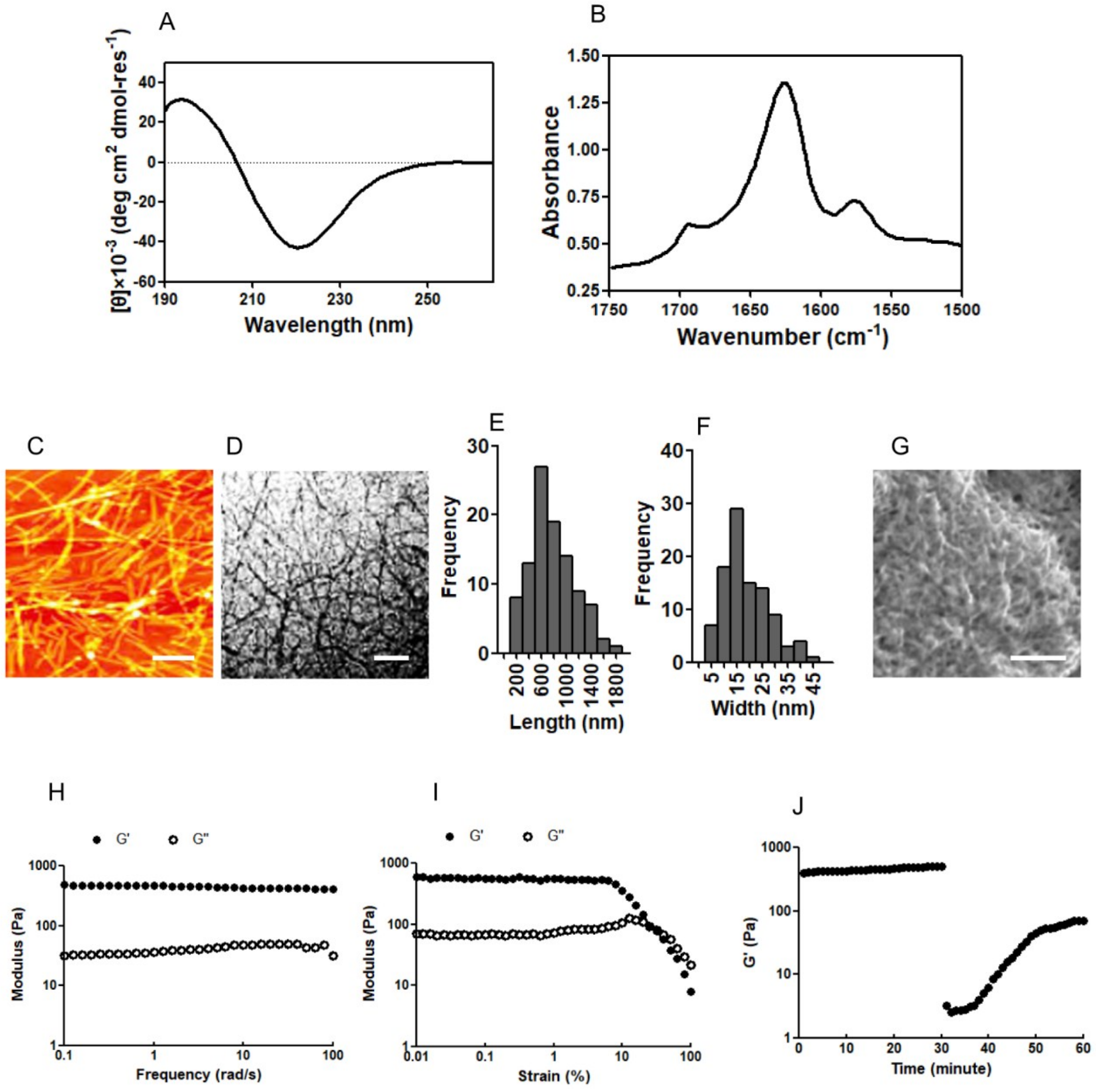

Fig18. Physicochemical characteristics of hydrogels after *A.Muciniphila* enzyme response and irradiation. (A) CD spectrum of hydrogels show the minima between 210 nm and 230 nm, suggesting the β-sheet formation. (B) FTIR spectrum of the amide I region showed the peaks of antiparallel β-sheet at 1630 cm^{-1} and 1695 cm^{-1}. (C) AFM images of peptide self-assembled peptide nanofibers, scale bars for 100 nm. (D) TEM images of peptide self-assembled peptide nanofibers, scale bars for 50 nm. The statistical results of (E) the lengths and the (F) width about self-assembled peptide nanofibers. (G) SEM images of hydrogels, scale bars for 500 nm. The G′ and G″with shear thinning detection of hydrogels through oscillatory shear rheology assays at (H) strain rates and (I) frequency oscillation. (J) The recovery detection of hydrogels from high shear rate.

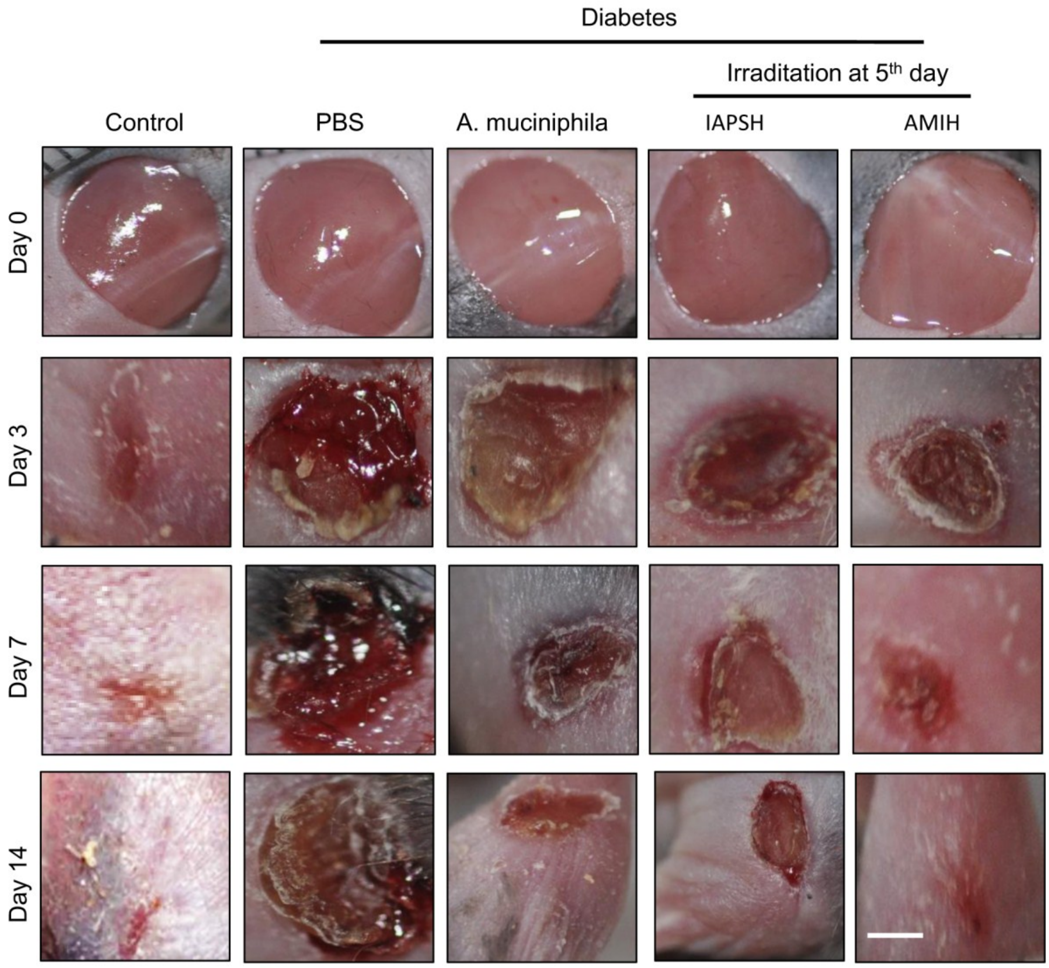

Fig19. The images of recovery wounds after different treatments at 0, 3, 7 and 14 days.

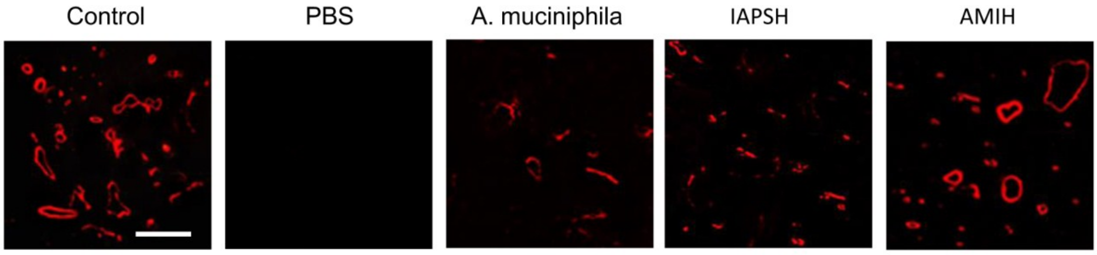

Fig20. CD31 staining for capillary detection.

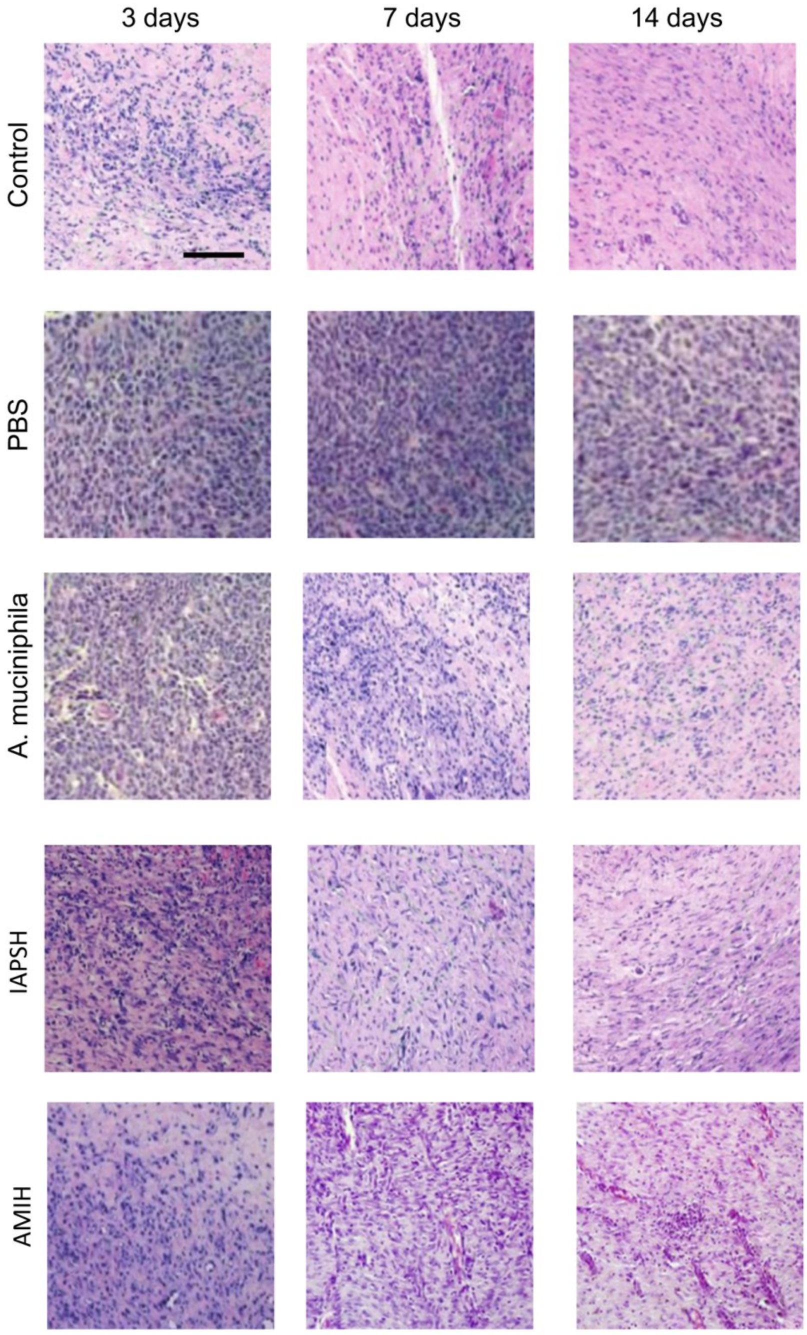

Fig21. Leukocytes infiltration detection though H&E staining.

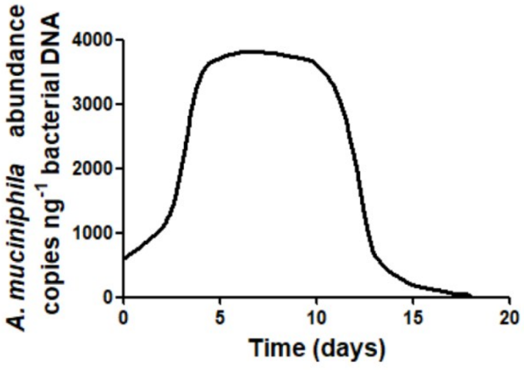

Fig22. The concentration of *A.Muciniphila* in ulcer tissue at different time by qPCR analysis.

Support materials and methods

Peptide synthesis and purification. All the peptides were synthesized by solid-phase peptide synthesis methodology as previous research reported.[1] All the coupling reagents and resin were purchased from Sangon Biotech (Shanghai, China). When the peptides were cleaved from the resin, rotoevaporation was used to remove the trifluoroacetic acid (TFA), and the residual TFA was triturated by cold diethyl ether and dried in the vacuum surrounding for 36 hours before purification. The purification was performed through HPLC assay according to the preparative reverse phase C-18 column with a linear water/acetonitrile gradient (0.05% TFA) at a flow rate of 15 ml/min. All the peptides were frozen and lyophilized, and stored at -20 °C, and then performed the mass spectrometry assay through MALDI-TOF MS (Brucker Daltonik GmbH, Bremen, Germany) to confirm the successfully synthesis of peptides.

2-amino-5-(4-(carboxymethoxy)-7-nitroindolin-1-yl)-5-oxopentanoic acid (ACNOA) synthesis. The ACNOA synthesis was performed according to the process of Fig7 (support information), which was reported by Ellis-Davies GC. [2] EtN=C=N$(CH_2)_3NMe_2$•HCl was added into MeCN and reaction for 40 hours at room temperature. And then NaOH was added into the MeOH solution for 3.5 hours at room temperature. And mixed the two solution, and then F_3CCO_2H was added into the mixture and reacted 4 hours at room temperature. Finally, $NaNO_3$ was added into the solution and reaction for 10 minutes at room temperature, and then purification and identification the products based on the HPLC, MALDI-TOF MS and Nuclear Magnetic Resonance (NMR) assays.

The modify of polypeptide skeleton (PPS). In order to obtain the modified PPS for further angiogenesis peptides and inflammatory memory peptides conjugation, PPS was modified by amine and azide (Fig4, support information) according to the previous reported method. [3] The product was purified by HPLC, and identified through MALDI-TOF MS.

Functional polypeptides conjugated with PPS. The inflammatory memory peptides and angiogenesis peptides were conjugated with PPS step by step as the protocol of previous report. [3] For the inflammatory memory peptides conjugation, the synthesized inflammatory memory peptides was added into the PPS solution, and the diisopropylamine (DIPEA) was used as the coupling reagent with the ratio of PPS: peptides: DIPEA=1:1:1.5, and reaction for 3 hours at room

temperature, then performing the subsequence couplings until the free amines was negative based on the ninhydrin test, collected the products then purification and identification, confirmed the inflammatory memory peptides were successfully conjugated. For the angiogenesis peptides conjugation, the angiogenesis peptides: inflammatory memory peptides-conjugated-PPS: CuSO4: sodium ascorbate=1.5:1:1:2, the mixture was dissolved in the dimethyl sulfoxide, and reaction at 40 °C for 24 hours and continuous stirring. Then dimethylformamide (DMF) was added into the solution as the ratio of 1:1, slowly added the products into the ice-cold diethyl ether and precipitated the products, then centrifugation with 3500 r/min for 20 min, remove the supernatant and the precipitates were collected, then purified and identified, obtaining the structures of functional peptides conjugated with PPS, as shown in Fig14 (support information).

Nanofiber hydrogel formation. The peptides IAPS were dissolved in the distilled water with the concentration of 2 wt%, adjusted to PH 7, and then IAPS solution was added into HBSS solution (Thermo, UAS), the volume ratio was 1:1, and $NaIO_4$ was added into the mixed solution with the concentration of 1 eq. to DOPA. After 24h oxidation, hydrophilic and hydrophobic binding, the hydrogel was formation and dialyzed by deionized water for 3 days. Obtained hydrogel was homogenized by sonication and then stored at 4°C.

Rheological analysis. All rheological researches were conducted through TA-AR200 rheometer (TA instruments, USA) using a 12 mm parallel plate with 1 mm gap height. 200 ul hydrogel solution was added into dialysis tubing (10 mm) and dialyzed for 24 h by deionized water, and then cut the gels with 1 mm slice and placed on the rheometer for the storage modulus (G′) and loss modulus(G′ ′) detection. Strain sweeps analysis was performed with the strain of 0.01% to 200% at a fixed oscillation frequency of 6.283 rad/s. Frequency sweeps analysis were performed at oscillation frequency of 0.1 rad/s to 200 rad/s under 1% strain. In the recovery experiments, 1% strain for 30 min, 100% strain for 60 s, 1 % strain for 30 min.

Transmission electron microscopy, atomic force microscope and scanning electron microscopy assays. The morphology of peptides nanofibers were observed through transmission electron microscopy (TEM) and atomic force microscope (AFM). For TEM, the samples were

added onto Quantifoil R1.2/1.3 holey carbon mesh on copper grids, and then 2.0% pH 7 phototungstic acid was used to negative staining for 10 min at room temperature, then observed and imaged the samples by H-7000FA TEM at 80-kV accelerating voltage (Hitachi, Tokyo, Japan). For AFM, samples were pipetted onto freshly cleaved mica plate and then fixed on the glass slide, 3 minutes later, rinsing the remained suspension through ultrapure water, and air-dried. The dimension FastScan AFM (Bruker, German) with FASTSCAN-A probe was used to obtain the AFM image. The morphology of hydrogel was detected through scanning electron microscopy (SEM), samples were dehydrated by gradient ethanol, and then dried and coated by gold (Denton, Moorestown, NJ), and the SEM images were obtained by FEI Quanta 400 ESEM (FEI Company, Hillsboro, OR).

Characterization of hydrogels detection. All the methods of hydrogels characterization detection were performed as previous research reported.[4] The circular dichroism (CD) with JASCO J-810 CD spectrometer and fourier transform infrared spectroscopy (FTIR) with Perkin-Elmer spectrum 100 FT-IR spectrometer assays were performed to confirm the formation of β-sheet structures of hydrogels. The mechanical properties of the hydrogels were detected through the oscillatory shear rheology on a TA-AR200 rheometer.

***A.Muciniphila* seeded and growth in hydrogels and tissues.** 1×10^{-3} TCID/ml (TCID = Median tissue culture infective dose, diluted in the PBS) *A.Muciniphila* was seeded in the hydrogels. The growth of *A.Muciniphila* was detected and recorded through quantitative polymerase chain reaction (qPCR) assay in 10μl system, which contain 0.5 unit FastStart Taq DNA polymerase (Roche, Mannheim, Germany) and0.5 μl EvaGreen (Tiangen, Beijing, China), and the primer of *A.Muciniphila* was forward 5′-CAGCACGTGAAGGTGGGGAC-3′; reverse 5′-CCTTGCGGTTGGCTTCAGAT-3′. The *A.Muciniphila* DNA extraction and qPCR assay were performed according to the previous protocol. [5]

Animal experiments. C57 mice were used in this study to establish the diabetes model. All the animal experiment procedures were approved by the Ethics Committee of the Third Military Medical University. 5-8 weeks old C57 mice (n=80, weight 20±4 g) were received intraperitoneally injected with 40 mg/kg streptozotocin (STZ) after 12 hours fasting every day, lasting 5 days to establish diabetes model. And the fasting blood glucose levels >16.7 mM, indicated that the diabetes model was successfully established. The diabetes mice were

anesthetized by 30 mg/kg sodium pentobarbital (10 mg/ml) through intraperitoneal injection. Ligation the terminal branches of the femoral artery and excision the skin tissue as 6×6 mm areas at the lateral thigh to establish the ischemic diabetic ulcer. Subsequently, the ulcers of mice were received the PBS (0.5ml, n=20), *A.Muciniphila* (1×10^{-3} TCID/ml, diluted in the PBS, 0.5ml, n=20), IAPSH (0.5ml, n=20) and AMIH (1×10^{-3} TCID/ml *A.Muciniphila* was seeded in the IAPSH, 0.5ml, n=20) treatments through subcutaneous injection. And the normal C57 mice (n=20) were performed the same operation procedure to set the control group.

Histological observation. The wounds recovery was recorded and photographed by D90 camera (Nikon Corporation, Tokyo, Japan) at days 0, 3, 7 and 14. The wounds areas were measured and calculated by Image Pro Plus 6.0 software (Media Cybernetics, Inc., Rockville, MD, USA) to detect the recovery of wounds. At day 3, 7 and 14, the tissues surrounding the wounds were collected and fixed with 4% paraformaldehyde and embedded in paraffin. Tissues were cut into 5-µm-thick sections and performed the hematoxylin and eosin (H&E) staining. Moreover, the sections collected from day 14 were performed the immunofluorescence assays for the angiogenesis detection through CD31 (Santa Cruz Biotechnology, TX, USA) staining. All the sections were observed and imaged through laser scanning confocal microscope (TCS-SP5; Leica Microsystems GmbH).

Extracellular glucose detection. The ulcers tissues were collected and gently grinded in the liquid nitrogen, and then centrifuged at 1000 r/min for 5 min, collected the supernatant which was the tissue liquid. The amount of extracellular glucose was detected through glucose hexokinase kit (Sigma, USA) using the Spectra Max 340PC spectrophotometer (Molecular Devices, USA) with the absorbance of 340 nm. All the samples were detected in triplicate and calculated out the average.

Statistical analysis. The data are expressed as the mean±S.D. Fisher´s test was used for the statistical analyses. The nonparametric Mann-Whitney rank-sum test was used to determine differences between samples. One-way ANOVA followed by Bonferroni post hoc testing were also used for specific experiment analyses. A threshold of $P<0.05$ was used to determine statistical significance.

Support reference

1. Dong H, Paramonov SE, Aulisa L, Bakota EL, Hartgerink JD. J Am Chem Soc. 2007 Oct 17;129(41):12468-72.
2. Ellis-Davies GC. Nat Protoc. 2011 Mar;6(3):314-26.
3. Pashuck ET, Duchet BJ, Hansel CS, Maynard SA, Chow LW, Stevens MM. ACS Nano. 2016 Dec 27;10(12):11096-11104.
4. Clarke DE, Pashuck ET, Bertazzo S, Weaver JVM, Stevens MM. J Am Chem Soc. 2017 May 31;139(21):7250-7255. \
5. Greer RL, Dong X, Moraes AC, Zielke RA, Fernandes GR, Peremyslova E, Vasquez-Perez S, Schoenborn AA, Gomes EP, Pereira AC, Ferreira SR, Yao M, Fuss IJ, Strober W, Sikora AE, Taylor GA, Gulati AS, Morgun A, Shulzhenko N. Nat Commun. 2016 Nov 14;7:13329.
